# Supplementary material for: Observation of super-ballistic Brownian motion in liquid
Source: Sci Adv. 2026 Feb 4;12(6):eaeb4579. doi: 10.1126/sciadv.aeb4579 (PMC12871451; doi:10.1126/sciadv.aeb4579)
Supplement: Supplementary file 1 — Supplementary Text Fig. S1 [file sciadv.aeb4579_sm.pdf]

Science Advances
AAAS

Supplementary Materials for

Observation of super-ballistic Brownian motion in liquid

Jason Boynewicz *et al.*

Corresponding author: Jason Boynewicz, jrb8338@my.utexas.edu

Sci. Adv. **12**, eaeb4579 (2026)
DOI: 10.1126/sciadv.aeb4579

This PDF file includes:

Supplementary Text
Fig. S1

5 Supplementary Text

5.0.1 High-Powered Detector and transfer function

We use a custom-built high-powered detector capable of withstanding 100 mW of laser power on each port of the detector. A second-order high-pass trans-impedance filter converts the photocurrent difference from the two detectors into a voltage signal. The high-pass filter built into the detector is necessary in order to apply the large incident powers onto the detector. Without the filter, low-frequency motion of the sphere and noise saturate the operational amplifier well before all incident laser power is used. To account for the filter, we empirically determine the transfer function of the detector by modulating the laser at known frequencies and monitoring the response of the detector. Since the laser itself has its own frequency-dependent response, the modulation was also monitored on a photodetector with a flat frequency response (Thorlabs PDB425C). We then fit a two-pole high-pass filter model to the empirical results. The resulting high-pass filter frequency response can be seen in Fig. S1.

Since the high-pass filter becomes singular at zero frequency, we face an inversion problem due to the low-frequency electronic noise sources in the system. To prevent large amounts of anomalous low-frequency noise while still inverting the high-pass filter, we apply a Tikhonov regularization scheme on the data. To do so, we Fourier transform our time trace, and then multiply the Fourier components by

$$\frac{H^*(f)}{|H(f)|^2 + |H(40)|^2}, \tag{S1}$$

where $H(f)$ is the transfer function of the high-pass filter in the detector. When the frequency approaches 40 Hz, the presence of the second term in the denominator prevents a singularity.

To ensure the regularization scheme has minimal impact on the extraction of the trap's physical parameters, we perform our same fitting procedure for several different cut frequencies between 20 Hz and 70 Hz. We find minimal dependence on the cut frequency with negligible changes in the radius and volts to meter conversion and changes in the strength of the trap on the order of 3.2%.

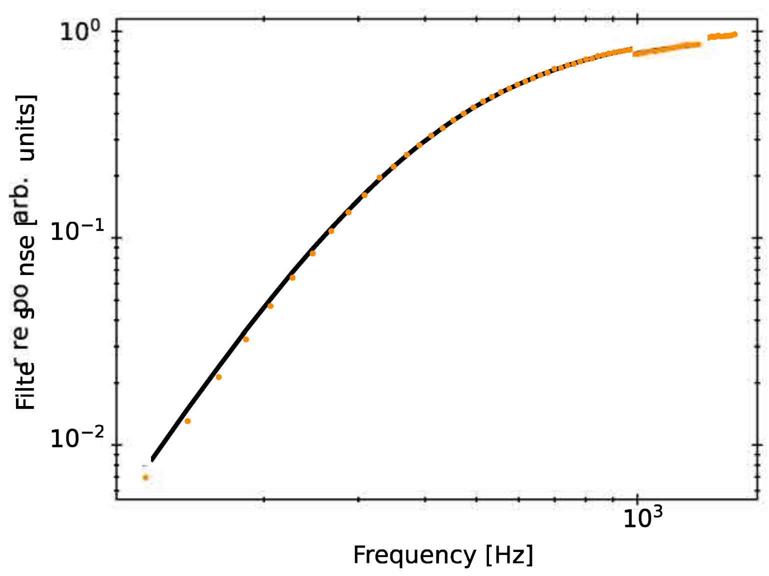

Figure S1: **Balanced detector characterization.** Plot of the amplitude response for the high pass filter on the high-powered balanced photodetector. The data are shown with the orange circles. The two-pole high pass filter fit is included with the solid black line.

5.0.2 Expansion of 0 Velocity MSD

For a free particle beginning at rest with no previous history of the fluid, Clercx and Schram (*31*) derived the VACF to be of the form

$$\langle v(t_1)v(t_2)\rangle = k_BT[\Lambda(|t_1 - t_2|) - M\Lambda(t_1)\Lambda(t_2)]\,, \tag{S2}$$

where

$$\Lambda(t) = \frac{1}{M(b-a)}[be^{b^2t}\,\mathrm{erfc}[b\sqrt{t}] - ae^{a^2t}\,\mathrm{erfc}[a\sqrt{t}]] \tag{S3}$$

$$z = 6\pi a^2\sqrt{\rho_f\eta} \tag{S4}$$

$$a = \frac{z}{2M}(1 + \sqrt{1 - \frac{4\gamma M}{z}}) \tag{S5}$$

$$b = \frac{z}{2M}(1 - \sqrt{1 - \frac{4\gamma M}{z}})\,. \tag{S6}$$

We expand this expression in powers of $\sqrt{t}$ to second order. Doing so yields an approximate short time expression for the VACF of

$$\langle v(t_1)v(t_2)\rangle \approx \frac{k_BT}{M^2}(\frac{2z}{\sqrt{\pi}}(\sqrt{t_1} + \sqrt{t_2} - \sqrt{|t_1 - t_2|}) + \frac{2}{M}(M\gamma - z^2)\min{[t_1, t_2]} - \frac{4z^2}{M\pi}\sqrt{t_1t_2})\,. \tag{S7}$$

The MSD can be found by integrating the VACF with respect to t_1 and t_2 yielding the result quoted in the main paper for the short time super-ballistic motion of

$$\text{MSD}[t] = \frac{2k_BT}{3M\tau_p}[\frac{12}{5}\sqrt{\frac{\tau_f}{\pi}}t^{5/2} + \beta t^3] \,. \tag{S8}$$

5.0.3 Solving the Hydrodynamic GLE

First, we separate the Basset-Boussinesq force at bound $t = 0$ and integrate the historical Basset-Boussinesq term by parts. The GLE becomes

$$M\ddot{x}(t) = -\gamma\dot{x}(t) - \gamma\sqrt{\frac{\tau_f}{\pi}}\frac{v(0)}{\sqrt{t}} + I(t) - \gamma\sqrt{\frac{\tau_f}{\pi}}\int_0^t \frac{\ddot{x}(\tau)}{\sqrt{t-\tau}}d\tau - Kx(t) + R(t)\,, \tag{S9}$$

where we denote the history force

$$I(t) = \gamma\sqrt{\frac{\tau_f}{\pi}}\int_{-\infty}^{0}\frac{\dot{x}(\tau)}{2(t-\tau)^{3/2}}d\tau\,. \tag{S10}$$

This procedure allows the boundary term to cancel the initial velocity term of the forward Basset-Boussinesq convolution in Laplace space. We then solve the equation by transforming to Laplace space to obtain

$$\begin{aligned} M[s^2\tilde{x}(s) - sx(0) - v(0)] = -\gamma[s\tilde{x}(s) - x(0)] - \gamma\sqrt{\frac{\tau_f}{s}}v(0) + \tilde{I}(s) \\ - \gamma\sqrt{\frac{\tau_f}{s}}[s^2\tilde{x}(s) - sx(0) - v(0)] - K\tilde{x}(s) + \tilde{R}(s)\,. \end{aligned} \tag{S11}$$

We solve for $\tilde{x}(s)$ in Laplace space and invert to get a function for x in the time domain and find

$$\begin{aligned} x(t) = Mx(0)A(t) + Mv(0)B(t) + \gamma x(0)B(t) + \gamma\sqrt{\tau_f}x(0)D(t) + \\ \int_0^t B(t-t')I(t')dt' + \int_0^t B(t-t')R(t')dt'\,, \end{aligned} \tag{S12}$$

where $A(t)$, $B(t)$, and $D(t)$ are defined in (*31*) as

$$A(t) = \frac{1}{M}\sum_{i=1}^{4}\frac{q_i^3\, e^{q_i^2 t}\ \text{erfc}(q_i\sqrt{t})}{\prod\limits_{j=1, j\neq i}^{4}(q_i - q_j)} \tag{S13}$$

$$B(t) = \frac{1}{M}\sum_{i=1}^{4}\frac{q_i\, e^{q_i^2 t}\ \text{erfc}(q_i\sqrt{t})}{\prod\limits_{j=1, j\neq i}^{4}(q_i - q_j)} \tag{S14}$$

$$D(t) = \frac{1}{M}\sum_{i=1}^{4} \frac{q_i^2\, e^{q_i^2 t}\ \mathrm{erfc}(q_i\sqrt{t})}{\prod_{j=1, j\neq i}^{4} (q_i - q_j)}, \tag{S15}$$

and q, indexed by i and j, runs over the four complex roots of the expression

$$Ms^2 + \gamma\tau_f s^{3/2} + \gamma s + K. \tag{S16}$$

5.0.4 History and Thermal Force statistical analysis

As a consequence of the fluctuation-dissipation theorem, we have shown in the main text that for a free particle

$$\langle R(t)v(0)\rangle = -\gamma\sqrt{\frac{\tau_f}{\pi}}\int_{-\infty}^{0} \frac{\langle v(0)v(t')\rangle}{2(t-t')^{3/2}} dt'. \tag{S17}$$

By assuming that $R(t)$ and $v(t)$ together form a high-dimensional multivariate Gaussian space, this result is sufficient to determine the expected mean trajectories and MSDs for preconditioned values for $v(0)$. As a direct consequence of Gaussianity, it follows that

$$\langle R(t)|v(0)\rangle = \langle R(t)v(0)\rangle\frac{v(0)}{\langle v^2(0)\rangle} = -\frac{v(0)}{\langle v^2(0)\rangle}\gamma\sqrt{\frac{\tau_f}{\pi}}\int_{-\infty}^{0} \frac{\langle v(0)v(t')\rangle}{2(t-t')^{3/2}} dt' \tag{S18}$$

$$\langle I(t)|v(0)\rangle = \frac{v(0)}{\langle v^2(0)\rangle}\gamma\sqrt{\frac{\tau_f}{\pi}}\int_{-\infty}^{0} \frac{\langle v(0)v(t')\rangle}{2(t-t')^{3/2}} dt'. \tag{S19}$$

These results are sufficient to demonstrate that the mean trajectory is agnostic to the details of the history; the conditioned thermal force hides the details of this past history from showing up in mean trajectories. We can apply the same analysis for the particle's MSD, where now we use the fact that

$$\langle R(t_1)R(t_2)|v(0)\rangle = \langle R(t_1)R(t_2)\rangle + \langle R(t_1)|v(0)\rangle\langle R(t_2)|v(0)\rangle - \frac{\langle R(t_1)v(0)\rangle\langle R(t_2)v(0)\rangle}{\langle v^2\rangle}. \tag{S20}$$

With these results for the conditional correlations, we can analyze the impact of the history on the MSD conditioned on the initial velocity of the particle. We will begin by analyzing the motion of a free particle with $x(0) = 0$ for simplicity, and solve for $\langle x(t_1)x(t_2)|v(0)\rangle$. Then the full MSD including the contributions of the history and the conditional thermal force becomes

$$\langle x(t_1)x(t_2)|v(0)\rangle = M^2 v(0)^2 B_f(t_1)B_f(t_2) + Mv(0)B_f(t_1)\int_0^{t_2} B_f(t_2-t')\langle R(t')|v(0)\rangle dt'$$
$$+ Mv(0)B_f(t_1)\int_0^{t_2} B_f(t_2-t')\langle I(t')|v(0)\rangle dt' + Mv(0)B_f(t_2)\int_0^{t_1} B_f(t_1-t')\langle R(t')|v(0)\rangle dt'$$
$$+Mv(0)B_f(t_2)\int_0^{t_1} B_f(t_1-t')\langle I(t')|v(0)\rangle dt' + \int_0^{t_1} B_f(t_1-t')\int_0^{t_2} B_f(t_2-t'')\langle R(t')R(t'')|v(0)\rangle dt''dt'$$
$$+\int_0^{t_1} B_f(t_1-t')\int_0^{t_2} B_f(t_2-t'')\langle R(t')I(t'')|v(0)\rangle dt''dt'$$
$$+\int_0^{t_1} B_f(t_1-t')\int_0^{t_2} B_f(t_2-t'')\langle I(t')R(t'')|v(0)\rangle dt''dt'$$
$$+\int_0^{t_1} B_f(t_1-t')\int_0^{t_2} B_f(t_2-t'')\langle I(t')I(t'')|v(0)\rangle dt''dt', \quad \text{(S21)}$$

where B_f is the Green's function for the free Brownian particle. The general form for a correlation function conditioned on the initial velocity is

$$\langle Y(t_1)Z(t_2)|v(0)\rangle = \langle Y(t_1)Z(t_2)\rangle + \frac{\langle Y(t_1)v(0)\rangle\langle Z(t_2)v(0)\rangle}{\langle v(0)^2\rangle}\left(\frac{v(0)^2}{\langle v(0)^2\rangle} - 1\right). \quad \text{(S22)}$$

Equation S18 and S22 allow us to evaluate the conditional correlation terms in equation S21, simplifying the conditioned MSD to

$$\langle x(t_1)x(t_2)|v(0)\rangle = M^2 v(0)^2 B_f(t_1)B_f(t_2) + \int_0^{t_1} B_f(t_1-t')\int_0^{t_2} B_f(t_2-t'')\langle R(t')R(t'')\rangle dt''dt'$$
$$+\int_0^{t_1} B_f(t_1-t')\int_0^{t_2} B_f(t_2-t'')\langle R(t')I(t'')\rangle dt''dt' + \int_0^{t_1} B_f(t_1-t')\int_0^{t_2} B_f(t_2-t'')\langle I(t')R(t'')\rangle dt''dt'$$
$$+\int_0^{t_1} B_f(t_1-t')\int_0^{t_2} B_f(t_2-t'')\langle I(t')I(t'')\rangle dt''dt'. \quad \text{(S23)}$$

The cancellation between terms results in dependence only on $v(0)$ and equilibrium correlations.

After using the definition of $I(t)$, we are left with a conditioned MSD involving the VACF, $C_{vv}(t)$, and the correlation $\langle R(t)v(\tau)\rangle$. By time translation invariance, this term is $\langle R(t-\tau)v(0)\rangle$. Using equation 17 we now have

$$\langle x(t_1)x(t_2)|v(0)\rangle = M^2v(0)^2B_f(t_1)B_f(t_2) + \int_0^{t_1} B_f(t_1-t')\int_0^{t_2} B_f(t_2-t'')\langle R(t')R(t'')\rangle dt''dt'$$
$$-\gamma^2\frac{\tau_f}{\pi}\int_0^{t_1} B_f(t_1-t')\int_0^{t_2} B_f(t_2-t'')\int_{-\infty}^0\int_{-\infty}^0 \frac{C_{vv}(\rho)}{4(t'-\tau-\rho)^{3/2}(t''-\tau)^{3/2}}d\rho d\tau dt''dt'$$
$$-\gamma^2\frac{\tau_f}{\pi}\int_0^{t_1} B_f(t_1-t')\int_0^{t_2} B_f(t_2-t'')\int_{-\infty}^0\int_{-\infty}^0 \frac{C_{vv}(\rho)}{4(t''-\tau-\rho)^{3/2}(t'-\tau)^{3/2}}d\rho d\tau dt''dt'$$
$$+\gamma^2\frac{\tau_f}{\pi}\int_0^{t_1} B_f(t_1-t')\int_0^{t_2} B_f(t_2-t'')\int_{-\infty}^0\int_{-\infty}^0 \frac{\langle v(\rho)v(\tau)\rangle}{4(t'-\rho)^{3/2}(t''-\tau)^{3/2}}d\rho d\tau dt''dt' \,. \quad \text{(S24)}$$

Consider the very last integral in this expression. We can rewrite it as

$$2\gamma^2\frac{\tau_f}{\pi}\int_0^{t_1} B_f(t_1-t')\int_0^{t_2} B_f(t_2-t'')\int_{-\infty}^0\int_{-\infty}^{\tau} \frac{\langle v(\rho)v(\tau)\rangle}{4(t'-\rho)^{3/2}(t''-\tau)^{3/2}}d\rho d\tau dt''dt' \,. \quad \text{(S25)}$$

Since in equilibrium the VACF is stationary, we can keep the value of the integral the same, as long as all the time differences are kept consistent, to achieve

$$2\gamma^2\frac{\tau_f}{\pi}\int_0^{t_1} B_f(t_1-t')\int_0^{t_2} B_f(t_2-t'')\int_{-\infty}^0\int_{-\infty}^0 \frac{C_{vv}(\rho)}{4(t'-\rho-\tau)^{3/2}(t''-\tau)^{3/2}}d\rho d\tau dt''dt' \,. \quad \text{(S26)}$$

This integral exactly cancels the other two terms involving the past dynamics of the particle. Therefore, for a free particle, we get perfect cancellation between the leftover history term and the information inferred about the stochastic force from knowledge of the initial velocity.

5.0.5 Inclusion of the trapping potential

It is not obvious that this cancellation with the thermal force works equivalently when the confining optical tweezer is added to the hydrodynamic Langevin equation. We repeat the analysis from the main text with the addition of the harmonic trap. The full Langevin equation becomes

$$M\dot{v}(t) = -\gamma v(t) - \gamma\sqrt{\frac{\tau_f}{\pi}}\frac{v(0)}{\sqrt{t}} + \gamma\sqrt{\frac{\tau_f}{\pi}}\int_{-\infty}^0 \frac{v(t')}{2(t-t')^{3/2}}dt' - \gamma\sqrt{\frac{\tau_f}{\pi}}\int_0^t \frac{\dot{v}(t')}{\sqrt{t-t'}}dt' + R(t) - Kx(t) \,. \quad \text{(S27)}$$

Repeating the analysis from the main text, we multiply by $v(0)$ and take the Laplace transform to get

$$\tilde{C}_{vv}(s)\left[Ms+\gamma+\gamma\sqrt{\tau_f s}+\frac{K}{s}\right] = MC_{vv}(0) - K\frac{\langle x(0)v(0)\rangle}{s} + \mathscr{L}\left[\langle R(t)v(0)\rangle + \gamma\sqrt{\frac{\tau_f}{\pi}}\int_{-\infty}^0 \frac{\langle v(0)v(t')\rangle}{2(t-t')^{3/2}}dt'\right] \,. \quad \text{(S28)}$$

Under the equilibrium assumption, we expect the cross correlation $\langle x(0)v(0)\rangle$ to be zero so that

$$\tilde{C}_{vv}(s) = \tilde{\mu}_K(s)\big[k_BT + \mathscr{L}[\langle R(t)v(0)\rangle + \gamma\sqrt{\frac{\tau_f}{\pi}}\int_{-\infty}^{0}\frac{\langle v(0)v(t')\rangle}{2(t-t')^{3/2}}dt']\big]\,, \tag{S29}$$

where $\tilde{\mu}_K(s)$ is the admittance for the trapped Brownian particle. The fluctuation-dissipation theorem then gives the identical result for the cross correlation $\langle R(t)v(0)\rangle$ as the case of a free particle. Since $\langle x(0)v(0)\rangle = 0$, the fact that $\langle R(t)v(0)\rangle$ takes the same value is sufficient to show that the same cancellation occurs for the trapped particle as the free particle. Thus, when calculating the MSD, terms involving correlations between the history, itself, the thermal force, and the velocity must all cancel out in the exact same way as above. Furthermore, we can still treat $\langle R(t)R(t')\rangle$ to have its equilibrium correlation properties.

To find the effect of the trapping potential on the MSD, we return to equation S12. Since we only condition on the velocity, we expect $\langle x^2(0)\rangle = \frac{k_BT}{K}$. The resulting expression for the conditioned position autocorrelation function becomes

$$\begin{aligned}\langle x(t_1)x(t_2)|v(0)\rangle &= k_BT[C(t_1)+C(t_2)-C(|t_2-t_1|)-MB(t_1)B(t_2)-KC(t_1)C(t_2)]+M^2v(0)^2B(t_1)B(t_2)\\ &+\frac{k_BT}{K}\big[M^2A(t_1)A(t_2)+\gamma^2B(t_1)B(t_2)+\gamma^2\tau_fD(t_1)D(t_2)+M\gamma[A(t_1)B(t_2)+B(t_1)A(t_2)]\\ &+M\gamma\sqrt{\tau_f}[A(t_1)D(t_2)+D(t_1)A(t_2)]+\gamma^2\sqrt{\tau_f}[B(t_1)D(t_2)+D(t_1)B(t_2)]\big]+\mathcal{F}(t_1,t_2)\,,\end{aligned} \tag{S30}$$

where $C(t)$ is defined by

$$C(t) = \frac{1}{K}+\frac{1}{M}\sum_{i=1}^{4}\frac{e^{q_i^2t}\,\mathrm{erfc}(q_i\sqrt{t})}{q_i\prod_{j=1,j\neq i}^{4}(q_i-q_j)}\,, \tag{S31}$$

and the final term, $\mathcal{F}(t_1,t_2)$, is given as

$$\begin{aligned}\mathcal{F}(t_1,t_2) &= \langle[Mx(0)A(t_1)+\gamma x(0)B(t_1)+\gamma\sqrt{\tau_f}x(0)D(t_1)][\int_0^{t_2}B(t_2-t')I(t')dt'+\int_0^{t_2}B(t_2-t')R(t')dt']\\ &+[Mx(0)A(t_2)+\gamma x(0)B(t_2)+\gamma\sqrt{\tau_f}x(0)D(t_2)][\int_0^{t_1}B(t_1-t')I(t')dt'+\int_0^{t_1}B(t_1-t')R(t')dt']\rangle\,.\end{aligned} \tag{S32}$$

Note that $\mathcal{F}(t_1, t_2)$ contains twelve cross terms involving the equilibrium correlation between the initial position and either the thermal force or the history force. Due to the separation in time scales between the trap dynamics and the velocity dissipation, we expect these terms' contribution to be small. We can calculate their contribution by following a procedure similar to the case of the velocity degree of freedom. As a consequence of linear response theory, we have that for the position autocorrelation function, $C_{xx}(t)$, the expression

$$-\frac{d^2}{dt^2}C_{xx}(t) = \langle v(t)v(0)\rangle = k_BT\mu_K(t) \tag{S33}$$

holds in equilibrium. Transforming this expression into the Laplace domain yields

$$-s^2\tilde{C}_{xx}(s) + sC_{xx}(0) + \dot{C}_{xx}(0) = k_BT\tilde{\mu}_K(s). \tag{S34}$$

By using $C_{xx}(0) = \frac{k_BT}{K}$ and $\dot{C}_{xx}(0) = 0$, this provides an expression for the equilibrium position autocorrelation in the Laplace domain of

$$\tilde{C}_{xx}(s) = \frac{k_BT}{K}\frac{Ms + \gamma + \gamma\sqrt{\tau_f s}}{Ms^2 + \gamma s + \gamma\sqrt{\tau s^3} + K}. \tag{S35}$$

Analogously to our argument for the thermal force-velocity cross correlation, we take our equation of motion, multiply both sides by $x(0)$, take the Laplace transform, and then take an ensemble average over equilibrium conditions. The resulting expression yields

$$\begin{aligned}\tilde{C}_{xx}(s) = &\frac{k_BT}{K}\frac{Ms + \gamma + \gamma\sqrt{\tau_f s}}{Ms^2 + \gamma s + \gamma\sqrt{\tau s^3} + K} \\ &+ \frac{1}{Ms^2 + \gamma s + \gamma\sqrt{\tau s^3} + K}\mathscr{L}[\langle R(t)x(0)\rangle + \gamma\sqrt{\frac{\tau_f}{\pi}}\int_{-\infty}^{0}\frac{\langle v(t')x(0)\rangle}{2(t-t')^{3/2}}dt'].\end{aligned} \tag{S36}$$

Utilizing our expression for the position autocorrelation function immediately yields the result quoted in the main text of

$$\langle R(t)x(0)\rangle = -\gamma\sqrt{\frac{\tau_f}{\pi}}\int_{-\infty}^{0}\frac{\langle v(t')x(0)\rangle}{2(t-t')^{3/2}}dt' \tag{S37}$$

.

The above expression immediately implies that $\mathcal{F}(t_1, t_2)$ is 0. Therefore, the equation used for the calculation of the theoretical curves for the velocity-conditioned MSD is arrived at using equation S30, neglecting $\mathcal{F}(t_1, t_2)$, and the definition of the MSD,

$$\text{MSD}(t) = \langle [x(0) - x(t)]^2\rangle. \tag{S38}$$

5.0.6 White Noise Langevin Equation

In the analysis of crossing times t_c, we compare the dynamics of the incompressible fluid with the Brownian motion of an equivalent particle in a forcing environment of white noise. In order to make this comparison, we require an analytic solution to this white-noise model system as well. The white-noise Langevin equation is defined as shown in the main text by

$$m\ddot{x}(t) = -\gamma\dot{x}(t) - Kx(t) + R(t) \tag{S39}$$

$$\gamma = 6\pi a\eta \tag{S40}$$

$$\langle R(t)R(t')\rangle = 2k_BT\gamma\delta(t-t')\,. \tag{S41}$$

This stochastic differential equation can be solved exactly for an arbitrary initial velocity and position. Doing so in the case of an overdamped trap yields the solution

$$x(t) = e^{-t/2\tau}(x(0)\cosh(\omega_1 t) + \frac{1}{\omega_1}(v(0) + \frac{1}{2\tau}x(0))\sinh(\omega_1 t) + \frac{\sqrt{2\gamma k_BT}}{m\omega_1}\int_0^t \sinh(\omega_1(t-s))e^{s/2\tau}dW_s)\,, \tag{S42}$$

where $\omega_1 \equiv \sqrt{-\omega_0^2 + \frac{1}{4\tau^2}}$, and dW_s is the increment of a Wiener process. From here, one can calculate the MSD for arbitrary initial conditions. We are interested in the case of a set initial velocity and thermally distributed initial position. For these initial conditions, the MSD is set by three terms associated with the initial spread of the position, the initial velocity, and the influence of the thermal force. The sum of these three effects then yields the full MSD, which takes the form

$$\begin{aligned}\mathrm{MSD}[t] &= \frac{2k_BTe^{-t/\tau}}{m\omega_1^2}\frac{-1-4\tau^2\omega_1^2(-1+e^{t/\tau}) + \cosh(2t\omega_1) + 2\tau\omega_1\sinh(2t\omega_1)}{8\tau^2\omega_1^2-2} \\ &+ \langle x(0)^2\rangle[1 - 2e^{-t/2\tau}(\cosh(\omega_1 t) + \frac{1}{2\tau\omega_1}\sinh(\omega_1 t)) + e^{-t/\tau}(\cosh^2(\omega_1 t) \\ &+ \frac{1}{4\tau^2\omega_1^2}\sinh^2(\omega_1 t) + \frac{1}{\omega_1\tau}\sinh(\omega_1 t)\cosh(\omega_1 t))] + v(0)^2\frac{1}{\omega_1^2}\sinh(\omega_1 t)^2 e^{-t/\tau}\,.\end{aligned} \tag{S43}$$

5.0.7 Uncertainty Estimates for Correlated Trajectories

When forming our ensembles of trajectories for a given initial velocity, nearby trajectories are necessarily correlated. Therefore, we cannot assume independence for all N of our experimental trajectories when calculating the uncertainty of our measurements. Usually, these correlations are

handled with the blocking method, as we have done when calculating our equilibrium MSD (*49*). However, since our trajectories are not pulled at regular intervals from the data set, it is not possible to directly implement this algorithm with the conditioned velocity ensembles. Instead, we note that if the sampled trajectories are far enough apart, the correlation between the trajectories decays away so that they are approximately independent. For short times, the dynamics are a consequence of the velocity degree of freedom, and therefore this correlation time is set by τ_p. At longer times, the dynamics are set by the interaction with the trapping potential and therefore correlations are primarily due to the position degree of freedom. The position correlation time is set by the trap strength and the damping acting on the fluid so that $\tau_K = \gamma/K$. To estimate the number of trajectories sufficiently separated to be considered statistically independent, we divide our time traces up into bins with width equal to τ_K. We then count how many bins contain the starting point for one of our velocity conditioned trajectories and use this to define the effective number of statistically independent trials for the experiment (N_{eff}). Our uncertainty estimate is given by

$$s_v = \frac{\sigma_v}{\sqrt{N_{eff}}} \tag{S44}$$

where s_v is our experimental uncertainty and σ_v is the experimental standard deviation across all of the trajectories for a given initial velocity. Note that for short lag times, this is a very conservative estimate, as the velocity de-correlates much faster than the position. For the longest lag times, it is also a conservative estimate, since it treats trajectories beginning in the same bin as perfectly correlated. We find that for all of the velocity conditioned MSD, mean trajectory, and variation around the mean trajectory graphs, $N_{eff}/N > 0.32$, so the effect on the uncertainty estimate is never greater than a factor of 1.8 times the uncertainty would be if we assumed all the traces were completely independent. Therefore, this conservative accounting of the correlation between trajectories has a limited effect on the experimental uncertainties.
